# The likely effects of thermal climate change on vertebrate skeletal muscle mechanics with possible consequences for animal movement and behaviour

**DOI:** 10.1093/conphys/coz066

**Published:** 2019-10-31

**Authors:** Rob S James, Jason Tallis

**Affiliations:** Research Centre for Sport, Exercise and Life Sciences, Coventry University, Coventry, UK

**Keywords:** Activity, force, locomotion, muscle, power, temperature

## Abstract

Climate change can involve alteration in the local temperature that an animal is exposed to, which in turn may affect skeletal muscle temperature. The underlying effects of temperature on the mechanical performance of skeletal muscle can affect organismal performance in key activities, such as locomotion and fitness-related behaviours, including prey capture and predator avoidance. The contractile performance of skeletal muscle is optimized within a specific thermal range. An increased muscle temperature can initially cause substantial improvements in force production, faster rates of force generation, relaxation, shortening, and production of power output. However, if muscle temperature becomes too high, then maximal force production and power output can decrease. Any deleterious effects of temperature change on muscle mechanics could be exacerbated by other climatic changes, such as drought, altered water, or airflow regimes that affect the environment the animal needs to move through. Many species will change their location on a daily, or even seasonal basis, to modulate the temperature that they are exposed to, thereby improving the mechanical performance of their muscle. Some species undergo seasonal acclimation to optimize muscle mechanics to longer-term changes in temperature or undergo dormancy to avoid extreme climatic conditions. As local climate alters, species either cope with the change, adapt, avoid extreme climate, move, or undergo localized extinction events. Given that such outcomes will be determined by organismal performance within the thermal environment, the effects of climate change on muscle mechanics could have a major impact on the ability of a population to survive in a particular location.

## Teaser

Changes in temperature, caused by climate change, can alter the amount of power an animal’s muscle produces, which could in turn affect that animal’s ability to catch prey or escape predators. Some animals may cope with such changes, but other species could undergo local extinction as a result.

## Introduction

Global average temperatures are rising at a rate of about 0.2°C per decade; the rate of temperature change is much greater in some regions, such as up to three times higher in the Arctic (IPCC, 2018). Global climate change is also initiating more frequent and more intense extreme weather events, such as heavy rainfall and heat waves causing floods and drought, respectively (IPCC, 2018). These acute and chronic changes in the local environment and threats to animal habitats brought about through climate change have, and continue to, alter the behaviour, geographical range, and survival of many animal species (Perry *et al.*, 2005; Chown *et al.*, 2010; Evans *et al.*, 2015; Beever *et al.*, 2017).

Many previous studies focusing on the effects of global climate change on animals have considered the effects of altered temperature. Such studies have demonstrated that changes in temperature have been linked with shifts in geographical range of species, effectively causing local extinctions. For example, the movement of most species of fish in the North Sea to more Northern and/or deeper, therefore colder waters (Perry *et al.*, 2005). There are many examples where explanation of such observed shifts, or prediction of likely future shifts, in species distribution is dependent on incorporating underlying temperature-induced physiological changes into the predictive models used (Chown *et al.*, 2010, Somero, 2012; Evans *et al.*, 2015). Some modelling studies have considered that rising temperatures could restrict the amount of time that individual animals can be active in their environment, thereby reducing the time available to partake in fitness-related behaviours (Evans *et al.*, 2015). However, more attention needs to be given to the effects of temperature on the actual performance of animals while they are active to improve modelling of the effects of climate change on animal species survival and distribution.

**Figure 1:**
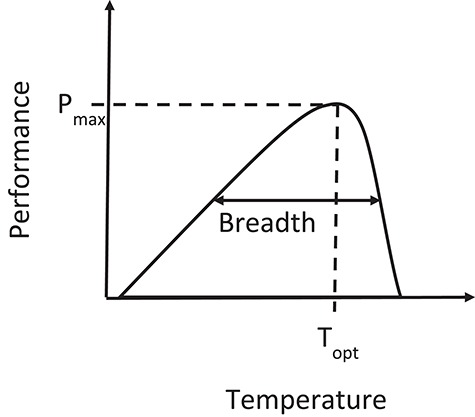
Theoretical performance curve showing the effect of temperature on performance. T_opt_ is the optimum temperature to maximize performance; P_max_ is the maximal performance; breadth is the performance breadth, which is the range of temperature over which performance is above a specified percentage of maximal performance.

Temperature affects the chemical and physical properties of animals and their environment, such as the rates of biochemical reactions within an animal and the density of fluids an animal moves through. Standard performance curves can be used to describe the change in performance with temperature, indicating the maximal performance, the optimal temperature for maximal performance (*T*_opt_), and the performance breadth, which is the range of temperature over which a specified level of performance can be attained (Fig. 1; Angilleta, 2009). For example, the reaction rates of metabolic enzymes are affected in a similar way to this standard curve with variation in *T*_opt_ of a specific enzyme between species or populations (Hochachka and Somero, 2002). Such enzymes exhibit relatively rapid declines in performance at higher than optimal temperature with inactivation and eventual denaturation occurring. Therefore, both increases and decreases in environmental temperature due to climate change could result in reductions in performance. Figure 1.

Given that animal performance is influenced by the mechanical performance of skeletal muscle, this review will focus on the acute and chronic effects of temperature change on skeletal muscle mechanics and consider how such effects could influence animal behaviour and survival as a result of climate change. Skeletal muscle mechanics has been shown to constrain aspects of animal performance that are important in some behaviours, such as sprinting, as used during an escape response, and bite force, as would be used during some aggressive behaviours. For example, previous studies have found strong correlations between individual variation in skeletal muscle mechanics or activity of metabolic enzymes in muscle, as a proxy of muscle mechanics, and variation in maximal sprint performance within a lizard species (Johnson *et al.*, 1993; Higham *et al.*, 2011). Variation in isolated iliotibialis, a leg extensor, muscle power output between related lacertid lizard species has been found to be strongly correlated, *r* = 0.77, with variation in sprint performance (Van Hooydonck *et al.*, 2014). A linkage has also been demonstrated between high performance in such muscle-powered activities and fitness, via longer-term survivorship or improved reproductive success (Miles, 2004; Lailvaux and Irschick, 2006; Husak *et al.*, 2008). Therefore, this review will also consider, where possible, how any temperature-induced alterations in muscle mechanics may impact locomotor performance and behaviour. Determining the existence of linkages between changes in skeletal muscle mechanics and effects on locomotor performance and behaviour is key to understanding the extent to which muscle mechanics could constrain locomotion and behaviour in a changing climate. Where possible, this review will differentiate between temperature effects on endotherms and ectotherms. Endotherms use heat from metabolism to regulate their core body temperature, often within a narrow range of temperatures, whereas the body temperature of ectotherms is dependent on their external environment and can undergo large daily and seasonal changes (Angilletta, 2009).

### Effects of acute temperature change on skeletal muscle mechanics

Changes in temperature can have profound effects on skeletal muscle mechanics. Studies, using skeletal muscle isolated from vertebrates, have generally demonstrated that up to an optimal, often relatively high, temperature, increased temperature causes greater force production, faster rates of force generation and relaxation, higher shortening speed, and enhanced power output (Bennett, 1984; Rall and Woledge, 1990; Syme, 2006; James, 2013). This general finding is consistent in both endotherms and ectotherms.

### Muscle mechanics during short-term activity

When a neurone stimulates a muscle cell, calcium is released from the sarcoplasmic reticulum into the muscle cytoplasm, increasing calcium concentration to initiate a chain of events that allow myosin to interact more strongly with actin (Jones *et al.*, 2004). Muscle force is produced via the interaction between myosin and actin, with myosin binding to actin to form cross-bridges that undergo a conformational change to produce force. These cross-bridges can be in low or high force–producing states (Ranatunga, 2018). When the neural stimulus ends, calcium is taken back into the sarcoplasmic reticulum, while parvalbumin also binds calcium in the cytoplasm and shuttles it to the sarcoplasmic reticulum, thereby reducing the concentration of calcium in the muscle cell; a reduction in calcium concentration decreases the number of interactions between myosin and actin, lowering muscle force to a ‘resting’ level (Berchtold *et al.*, 2000). Therefore, greater calcium concentration in the cytoplasm causes higher numbers of cross-bridges to form and greater force production, whereas more rapid calcium release into the cytoplasm leads to faster force generation and more rapid calcium uptake from the cytoplasm causes faster muscle relaxation.

**Figure 2:**
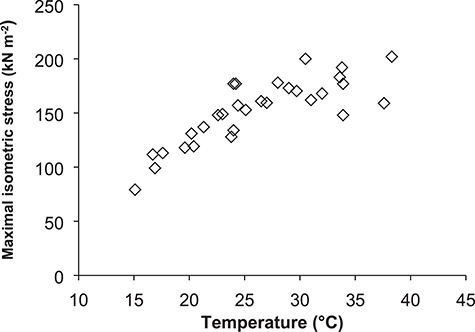
Effect of temperature on the maximal isometric stress (force normalized to muscle cross-sectional area) generated by isolated mouse diaphragm muscle. Each data point represents the maximum stress generated by one muscle at that temperature. Eight muscles were subjected to four different temperatures each. Temperature was randomized for each muscle. Based on data presented in James *et al.* (2015).

Isolated skeletal muscle mechanics has often been determined using isometric studies, whereby the muscle is activated while kept at an overall constant length (Josephson, 1993; Caiozzo, 2002). The maximal isometric force that a skeletal muscle can produce increases as temperature rises (Bennett, 1984; Rall and Woledge, 1990; Syme, 2006; James, 2013). However, at higher temperatures, this change in force is usually relatively low, and above the optimal temperature, a gradual reduction in force occurs (Fig. 2). For example, a comparison of maximum isometric force produced by single fibres from species of ectothermic fish from the Antarctic, North Sea, and Central Africa demonstrated that the muscle of each fish produced maximal force at temperatures around those that occurred in their natural environment, with decreases in force at lower and higher temperatures, such that each species outperformed the other species when in its own physiological temperature range (Altringham and Johnston, 1986). Skeletal muscle in endotherms has also been found to show high thermal sensitivity of isometric force production outside of physiological temperature ranges, regardless of whether the muscle is from the body core, where temperature is relatively constant, or the periphery of the body, such as found in diaphragm (core) and soleus (peripheral) muscle isolated from laboratory mice, *Mus musculus* (James *et al.*, 2015), or in muscle from different regions of endothermic fish (Altringham and Block, 1997; Bernal *et al.*, 2005; Donley *et al.*, 2012). As temperature rises, there is no change in the number of cross-bridges that form (myosin heads attached to actin binding sites) within skeletal muscle, but there is an increase in the proportion of cross-bridges that are in a high force producing state, thereby enhancing muscle force generation (Bershitsky and Tsaturyan, 2002; Decostre *et al.*, 2005; Colombini *et al.*, 2008; Ranatunga, 2018). Figure 2.

During isometric actions, temperature does not just affect the maximal amount of force produced. Rates of force generation and relaxation, during isometric activities, increase as temperature rises (Ranatunga, 1982; John-Alder *et al.*, 1988; Swoap *et al.*, 1993; Altringham and Block, 1997; De Ruiter and De Haan, 2000; Wilson *et al.*, 2000; Herrel *et al.*, 2007; James *et al.*, 2015). Data from both ectothermic and endothermic species indicate that the rate of change decreases as the optimal temperature for maximal mechanical performance of muscle is approached. Changes in rates of force generation and relaxation could have important influences on whole animal performance; in some species, they may influence the stride frequency that an animal can attain (Johnson *et al.*, 1993; see discussion below on temperature effects on work loops). Increased temperature raises myofibrillar ATPase activity and the rate at which parvalbumin binds calcium, thereby increasing rates of isometric force generation and relaxation, respectively (Barany, 1967; Stein *et al.*, 1982; Hou *et al.*, 1992). For example, as temperature increased from 5°C to 35°C, there was a high correlation (*r* = 0.99) between change in rate of myofibrillar ATPase activity and rate of force generation in skinned psoas muscle fibres from rabbit, *Oryctolagus cuniculus* (Brenner and Eisenberg, 1986).

Isolated muscle power output has traditionally been assessed during force–velocity experiments, whereby muscle is activated to produce force while at constant length and then shortened at a constant velocity or at a constant force (Josephson, 1993; Caiozzo, 2002). Such controlled shortening actions are repeated numerous times on a muscle preparation to determine the relationship between force output and shortening velocity in that muscle. Power output is calculated as force generated multiplied by shortening velocity. During such force–velocity experiments, performance of skeletal muscle generally improves as temperature rises, with increases in estimates of maximum shortening velocity and power output (Hill, 1938; Ranatunga, 1982; Marsh and Bennett, 1985; Johnston and Gleeson, 1987; Coughlin *et al.*, 1996; Ranatunga, 1998). Again, the rate of change decreases as temperature approaches that normally used during locomotion. Notably, Olberding and Deban (2017) demonstrated, during force–velocity experiments, that frog, *Osteopilus septentrionalis*, plantaris muscle work output and shortening velocity exhibited much lower thermal sensitivity during low force, as would be used during routine movement or postural control, than high force contractions, such as would be used during maximal activities.

**Figure 3:**
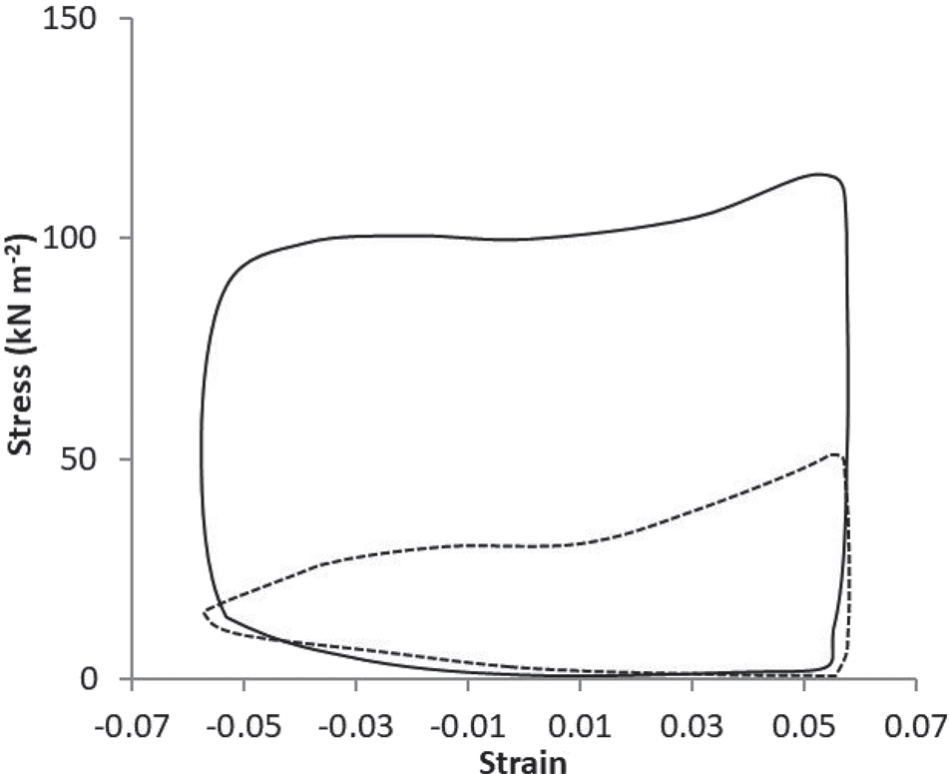
Typical effects of temperature on work loop shape. Mouse soleus work loop shapes at maximal power output at 15.3°C (broken line) and 37.4°C (solid line) in the same muscle preparation. Maximal power output was produced at a length change cycle frequency of 1 Hz at 15.3°C and 5 Hz at 37.4°C. Force was normalized to muscle cross-sectional area to calculate muscle stress and muscle length change was normalized to muscle length to calculate strain. Based on data presented in James *et al.* (2015). These work loop shapes demonstrate that at the higher temperature, there was more rapid force generation, greater maximal force, improved maintenance of force during shortening (likely to be at least partly due to an increased maximal shortening velocity, thereby altering the force–velocity relationship), and more rapid force relaxation.

Work loop experiments were developed to allow closer *in vitro* simulation of the type of muscle actions that occur *in vivo* during power-producing activities (Josephson, 1993; Caiozzo, 2002). The force generated by the muscle is plotted against the length change to produce a work loop, the area of which represents the work done during a length change cycle (Fig. 3). The power generated by the muscle can be determined as the sum of work done divided by the time taken to do that work. As temperature rises up to normal body temperature, there is an increase in work loop power output, in muscle isolated from amphibians, fish, mammals, and reptiles (Johnson and Johnston, 1991; Swoap *et al.*, 1993; Altringham and Block, 1997; Rome *et al.*, 1999; Donley *et al.*, 2007; Herrel *et al.*, 2007; Seebacher and James, 2008; James *et al.*, 2012; Seebacher *et al.*, 2014; James *et al.*, 2015). As temperature rises, work loop power output can be enhanced by a combination of increasing the maximal shortening velocity of the muscle such that maximal power output is achieved at a higher cycle frequency and by increasing the area of the work loop via the following: (i) reducing the time taken to generate force and to relax; (ii) enhancing the muscle's ability to produce force while shortening; and (iii) a reduction in the passive resistance to lengthening (Fig. 3; James, 2013). Thermal sensitivity of muscle power output varies between species with, in general, higher thermal sensitivity in skeletal muscle from endotherms. Importantly, power output determined by work loops also indicates that increased temperature above, or below, that normally experienced can result in a decrease in power output, such that the optimal temperature for skeletal muscle power output generally seems to be about the normal active body temperature. Figure 3.

In endotherms, there is evidence that some discrepancies in thermal sensitivity between skeletal muscles may relate to differences in location of each muscle in the body that result in variation in the range of temperature experienced. Skeletal muscle from the core of the body is maintained at a more narrow range of temperatures and has been found to have greater thermal sensitivity than muscle from the periphery in fish exhibiting regional endothermy (Altringham and Block, 1997; Bernal *et al.*, 2005; Donley *et al.*, 2007, 2012) with comparably little difference found in mouse (James *et al.*, 2015). Work loop power output showed higher thermal sensitivity in muscle isolated from the deep endothermic core of yellow fin tuna, *Thunnus albacares*, than from the more superficial region of skeletal muscle in this fish (Altringham and Block, 1997). A comparison between bat, *Carollia perspicillata*, wing muscle (extensor carpi radialis longus) and mouse, *M. musculus*, limb muscle (extensor digitorum longus) demonstrated that isometric force generation and relaxation times and maximal shortening velocity had lower thermal sensitivity, below core body temperature, in the bat wing muscle, which is likely to be subjected to much higher temperature ranges during flight than would occur in mouse limb muscle (Rummel *et al.*, 2018). Therefore, it seems that muscles subjected to a more narrow range of temperatures, as would be expected in core body muscles in endotherms, can become specialized to produce higher mechanical performance over a narrow thermal range, while having higher thermal sensitivity, as predicted by theory of a generalist–specialist continuum (Angilleta, 2009; Angilletta *et al.*, 2010). However, such differences may be dependent on species and the mechanical property measured.

### Muscle mechanics during sustained activity

In some fitness-related animal behaviours, high performance cannot be attributed to an ability to produce bursts of high muscle force or power but is instead due to an ability to sustain muscle activity over a series of actions. Prolonged muscle and locomotor performance is affected by fatigue. Skeletal muscle fatigue is defined as a reversible progressive reduction in contractile function (Allen *et al.*, 2008).

**Figure 4:**
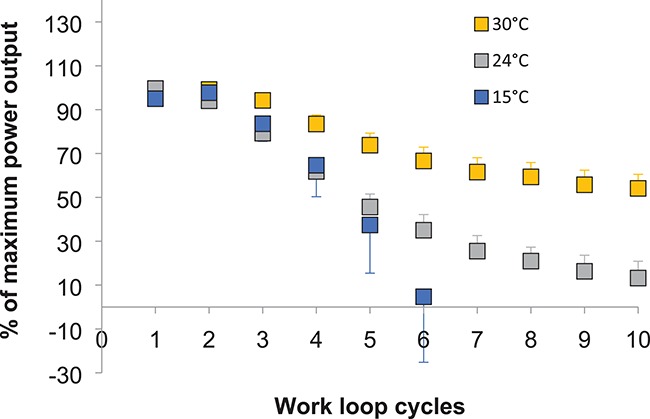
Fatigue resistance of power production improved as test temperature increased during a series of work loops in *X. tropicalis* iliotibialis muscle. Orange, grey, and blue symbols represent 30°C, 24°C, and 15°C data, respectively, for individuals housed at 24°C. Data represented as mean ± sem, *n* = 8, plotted as a percentage of the maximum power output produced by each individual. Based on data presented in James *et al.* (2012).

While other mechanical variables show a typical trend of a temperature-induced improvement in performance, this is not consistently shown in the literature considering sustained muscle activity. Work by James *et al.* (2012) reported that the endurance of iliotibialis muscle isolated from *Xenopus tropicalis*, measured via a change in maximal work loop power over a protocol of repeated activations, increased with temperature (Fig. 4). A temperature-induced improvement in fatigue resistance has been reported in previous work using both protocols of repeated isometric tetani and cycles of active shortening (Roots *et al.*, 2009). Conversely, there is conflicting evidence that increased temperature has limited effects on fatigue resistance (Place *et al.*, 2009, Nocella *et al.*, 2013) or may even cause fatigue to occur more quickly (Segal *et al.*, 1986). Figure 4.

Ambiguity in findings make it difficult to make comparisons between studies, and such disparity is likely to arise, at least in part, from methodological discrepancies, including whether the test temperature was close to normal body temperature, relevance of the fatigue protocol with respect to real-world function, and the complexity around aetiology of fatigue. As an example, previous work defines fatigue by an arbitrary decline in performance relative to a pre-fatigue maximal (Roots *et al.*, 2009) or over an arbitrary number of predefined activations (ranging from 10 to 105 activations) (James *et al.*, 2012, Nocella *et al.*, 2013, Place *et al.*, 2009) and is either measured during maximal or sub-maximal activation, force, or power-generating conditions. Fatigue occurs as a two-phase decline, whereby the initial rapid decline in muscle function may take longer and be attenuated at higher temperatures, but an increased temperature may cause performance to decline more rapidly in the latter phase where the loss of force occurs more slowly (Roots *et al.*, 2009, Nocella *et al.*, 2013). These distinct phases are not always considered in previous work and may be apparent without a temperature-induced change in the end point of fatigue (Nocella *et al*., 2013).

Interestingly, the previously outlined temperature-induced increase in fatigue resistance reported by James *et al.* (2012) did not translate to whole-organismal endurance in the same species, *X. tropicalis*, where jump performance was decreased either side of an optimal temperature (Herrel and Bonneaud, 2012). This may indicate that exertion capacity may be limited by factors external to the muscular contractile elements (Herrel and Bonneaud, 2012) or that the chosen fatigue protocol may not relate closely enough to the *in vivo* locomotor demands of the whole organism. Studies using relatively long duration fatigue protocols may not accurately represent movements involved in feeding and burst locomotion, which are performed using single or small numbers of muscle activations (Olberding and Deban, 2017, Wilson *et al.*, 2002). Burst performance, which is the result of a small number of high-force muscle activations, has been documented to be important for performance (Segre *et al.*, 2015, Bennett, 1994) and survival (Watkins, 1996), so a reduction in phase one of the fatigue response with increasing temperature may be beneficial to some species in more burst like, shorter, periods of prolonged muscular activity.

The aetiology of fatigue is complex, is species and muscle specific, and is also temperature sensitive (Allen *et al.*, 2008, Fitts, 2008, James *et al.*, 2012, Debold *et al.*, 2016). For example, skeletal muscle fatigue has, at least in part, been attributed to accumulation of inorganic phosphate and hydrogen ions, which have been shown to directly inhibit interactions between myosin and actin, during the cross-bridge cycle, and to indirectly cause lowered myofibrillar Ca^2+^ sensitivity, such that less force is produced at a specific calcium concentration, and there is a reduction in sarcoplasmic reticulum Ca^2+^ release (Allen *et al.*, 2008, Fitts, 2008; Debold *et al.*, 2016). There is evidence to indicate that increasing temperature may reduce the effects of high concentrations of inorganic phosphate and hydrogen ions on muscle force production (Coupland *et al.*, 2001, Debold *et al.*, 2016), which would support evidence showing that temperature can, at least in some muscles, improve fatigue resistance.

A further important aspect of fatigue is the mechanical efficiency of muscle activity, which has also been demonstrated to be temperature sensitive. Data by Edwards *et al.* (1972) indicated that increasing temperature reduced the mechanical efficiency of isometric contractions in human quadriceps muscle *in situ*, such that higher temperatures elicited greater energy cost and more rapid fatigue while undertaking the same activity. However, it has been more recently demonstrated that temperature effects on mechanical efficacy can be related to contractile velocity and, more specifically, a rightward shift in the efficiency–velocity relationship, whereby low-velocity muscle action may be less efficient at higher temperatures due to an increased speed of cross-bridge cycling, and at higher temperature, mechanical efficacy increases (Ferguson *et al.*, 2002).

Despite the outlined challenges with summarizing the current literature, it is likely that the effect of temperature on prolonged muscle activity is species specific. A disparity in subsequent locomotor performance between predator and prey as a result of changing temperature may affect the survival and abundance of a particular species. For example, Allan *et al.* (2015) found that exposure to an elevated temperature influenced predator–prey interactions of two common reef fish. The piscivorous dottyback (*Pseudochromis fuscus*) demonstrated increased attack speed, while the planktivorous damselfish (*Pomacentrus wardi*) had decreased escape speeds and distances leading to increased predation rates. Other work has demonstrated that temperature effects on predator–prey relationships can, in some instances, also favour prey (Abrahams *et al.*, 2007).

### Can the effects of acute temperature change on skeletal muscle mechanics constrain locomotion and behaviour?

A key question as to whether changes in skeletal muscle performance are important to survival of individuals or species is whether there is a direct link between variation in the mechanical performance of skeletal muscle, changes in locomotor performance, and subsequent alterations in behaviour. Modelling conducted by Seebacher *et al.* (2015) demonstrated that individual variation in the mechanical performance of isolated skeletal muscle could partially explain observed differences in the overall swimming performance between those individual zebrafish, which in turn explained the time a fish was active in a novel environment and in turn an individual’s boldness in approaching a novel object. Thus, simplistically, longer, lower-weight fishes that had muscle that produced higher force at a quicker rate were more likely to have higher sustained and sprint swimming performance, such that they were more likely to spend more time active and to cover a greater distance in the behavioural test in the novel environment and were in turn more likely to show higher boldness in approaching a novel object (Seebacher *et al.*, 2015). These findings suggest that variation in the mechanical performance of skeletal muscle can explain differences observed between individuals in locomotor performance and, in turn, behaviour. Thus, could effects of temperature on muscle mechanics and locomotion alter behaviour?

One of the best examples of the effects of acute temperature on locomotor performance and behaviour is the fight or flight response observed in some lizard species. At higher temperatures, such lizards will tend to flee from perceived predation risk. However, as environmental, and hence body, temperature decreases, it becomes increasingly likely that an individual will act aggressively and may attempt to bite the predator rather than run away (Hertz *et al.*, 1982; Crowley and Pietruszka, 1983; Mautz *et al.*, 1992). Sprint performance in lizards has high thermal sensitivity, with maximal and near maximal performance occurring over a narrow range of relatively high temperatures (Bennett, 1980; Huey and Kingsolver, 1989; Swoap *et al.*, 1993; Herrel *et al.*, 2007). In contrast, the maximal bite force produced by *Trapelus pallida*, a species of agamid lizard that exhibits this temperature-related shift between fight and flight behaviour, is relatively independent of temperature (Herrel *et al.*, 2007). Herrel *et al,* (2007) demonstrated that the thermal sensitivity of the mechanical performance of muscle could explain the effects of temperature on bite force and running performance measured in this species. Isolated jaw muscle from *T. pallida* exhibited almost no change in maximal isometric force production between test temperatures of 20°C and 40°C, whereas caudofemoralis, a large muscle that retracts the femur, reduced in maximal isometric force by 20% between 35°C and 20°C (Herrel *et al.*, 2007). However, while isometric force production is important for jaw muscles during biting, the caudofemoralis muscle is likely to act primarily to produce power during sprinting. The maximal power produced by isolated caudofemoralis muscle during work loops decreased by more than 40% between 35°C and 20°C (Herrel *et al.*, 2007); other previous work on the desert iguana, *Dipsosaurus dorsalis*, has also found that isolated limb muscle power output, determined by work loops, has a high thermal sensitivity (Swoap *et al.*, 1993). Overall, previous findings indicate that at least in these species studied, the acute thermal effects on the mechanical performance of skeletal muscle cause large changes in sprint performance but not bite force, due to the type of muscle activity being undertaken, as well as the differing intrinsic properties of the muscles involved. These thermal effects on sprint performance drive the observed shift from escape behaviour to aggressive behaviour at lower temperature in such lizard species. However, in some species of lizard, such as the Jamaican Grey anole *Anolis lineatopus*, individuals start their escape response from predators sooner at lower temperatures, rather than using any form of defensive behaviour, presumably as a different strategy to account for lower mechanical performance of limb muscle at reduced temperatures (Rand, 1964).

Michelangeli *et al.* (2018) demonstrated that behaviour of individuals of delicate skink, *Lampropholis delicate*, was linked to thermal sensitivity of performance such that a distinct ‘hot’ group of individuals had higher preferred body temperature, lower breadth of selected body temperature, higher optimal performance temperature, and lower thermal performance breadth than the ‘cold’ group; the hot group, when at a common temperature, spent more time active, spent more time exploring and less time hiding when in a novel environment, and had faster maximal sprint speeds than the cold group. These findings suggest that thermal physiology can constrain behaviour in predictable ways, affecting the way that individuals use niches within a habitat.

While the published literature is relatively sparse, it seems likely that colder or hotter environments will affect the mechanical performance of skeletal muscle and could in turn influence aspects of animal performance and behaviour, but further work is needed to better elucidate such relationships. What is clear is that some species are able to respond to changes in environmental temperature by undergoing acclimation of physiological processes or spending time in a ‘dormant’ state.

### Chronic responses to temperature—acclimation and dormancy

As environmental temperature alters over time, individuals may be able to continue to be active and survive at that temperature, if their thermal sensitivity is sufficiently low, or may undertake a physiological response, such as acclimation or dormancy, or may be forced to move to an environment with a more favourable temperature.

### Thermal acclimation

**Figure 5:**
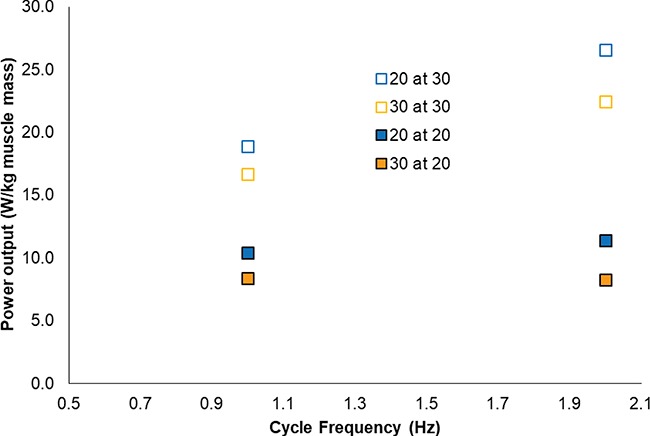
Power output increased with temperature and was greater in cold-acclimated animals (20°C, blue symbols) than warm-acclimated animals (30°C, orange symbols), regardless of test temperature (open symbols at 30°C, closed symbols at 20°C) or frequency of length change cycles, in caudofemoralis muscle isolated from saltwater crocodile. Data represents mean ± sem, *n* = 8. Based on data presented in Seebacher and James (2008).

Acclimation responses occur in many species that are subjected to relatively large, seasonal changes in temperature and involve physiological changes to improve aspects of performance at the seasonal temperature. In many, but not all, cases acclimation responses can be beneficial, enabling changes in the thermal sensitivity of physiological processes to effect seasonal compensation in performance, such as to improve the mechanical performance of skeletal muscle and consequently locomotor performance at relatively low temperatures (Johnston and Temple, 2002; Wilson and Franklin, 2002; Seebacher, 2005; Angilletta, 2009). For example, muscle power output increased with temperature in caudofemoralis (the main muscle to power swimming) isolated from saltwater crocodiles, *Crocodylus porosus*, regardless of acclimation temperature (Seebacher and James, 2008; Fig. 5). However, cold-acclimated (20°C) individuals had a higher rate of caudofemoralis force generation and relaxation than those that were warm acclimated (30°C), enabling higher work loop power output in cold-acclimated muscle when tested at either acclimation temperature (Seebacher and James, 2008). In this study, the thermal sensitivity of myofibrillar ATPase activity of caudofemoralis muscle decreased with cold acclimation, providing at least some explanation for the results obtained (Seebacher and James, 2008). Glanville and Seebacher (2006) found, in much smaller individuals of the same species, that cold acclimation (20°C) enabled individuals to achieve higher maximum sustained swimming performance (*U*_Crit_) than warm-acclimated (30°C) individuals when tested at the cold acclimation temperature, the reverse being true at 30°C. Therefore, in the saltwater crocodile, the cold acclimation of skeletal muscle helps explain the compensation in swimming performance seen at lower temperatures in cold-acclimated animals. In another example, acclimation of red (slower, more aerobic) muscle mechanics and swimming performance was compared between rainbow smelt, *Osmerus mordax*, and rainbow trout, *Oncorhynchus mykiss*, that tend to encounter relatively higher and lower ranges of temperature, respectively, over a year (Shuman and Coughlin, 2018). In both species, red muscle isolated from cold-acclimated individuals had faster rates of isometric force generation and relaxation than warm-acclimated individuals at the two test temperatures used, representing relatively cold and mid-range environmental temperatures, although this effect was greater in smelt (Shuman and Coughlin, 2018). In work loop studies, there was no significant effect of acclimation group on maximum skeletal muscle power output (Shuman and Coughlin, 2018). In both species, cold-acclimated fish showed higher maximum steady swimming speed, than warm-acclimated fish, at the mid-range test temperature, the difference between acclimation groups being much greater in smelt, the species that are subjected to a higher annual range in temperature (Shuman and Coughlin, 2018). Figure 5.

Usually, acclimation occurs when the seasonal change in temperature is predictable and large when compared to daily variation in temperature. In contrast, species that live in environments with relatively high daily variation in temperature may have lower thermal sensitivity enabling them to perform reasonably well over a comparatively broad range of temperatures (Angilletta, 2009). For example, amphibian tadpoles, which often live in relatively small volumes of water that can be subjected to high daily thermal variability, on the whole seem unable to acclimate, instead having comparably low thermal sensitivity such that locomotor performance is near maximal across a broad range of temperatures (Niehaus *et al.*, 2011). In contrast, Wilson and Franklin (1999) demonstrated that one species of tadpole, the striped marsh frog, *Limnodynastes peronei*, when acclimated to 10°C achieved about 50% greater maximum swimming velocity and acceleration, at a relatively low temperature of 10°C, in comparison to those acclimated to 24°C. When acclimation period at 10°C was increased from 6 weeks to 8 months, there was also a marked reduction in swimming performance at 24°C (Wilson and Franklin, 1999). Niehaus *et al.* (2011) tested whether daily thermal variation around a mean acclimation temperature caused differences in thermal sensitivity of performance in striped marsh frog. They found that swimming performance and skeletal muscle lactate dehydrogenase (a marker enzyme for anaerobic metabolic capacity in skeletal muscle) activity showed the same thermal sensitivity across a temperature range of 14–34°C regardless of the acclimation treatment, demonstrating that large daily thermal variation does not affect acclimation response in some species (Niehaus *et al.*, 2011).

**Figure 6:**
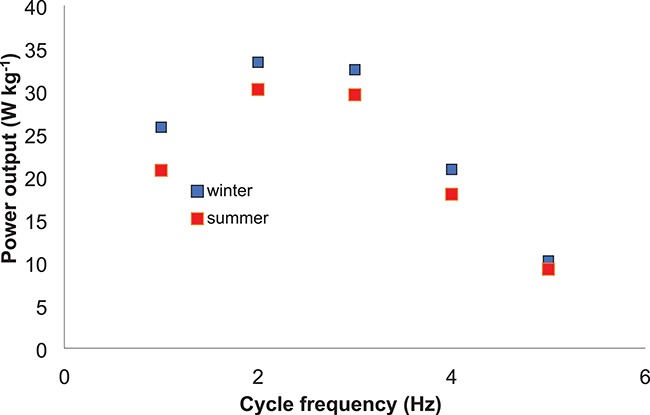
There was no significant difference in the soleus muscle power output–cycle frequency relationship between muscle removed from 13-lined ground squirrel in the summer (red) compared with that removed from those that had undergone 3 months of torpor in winter (blue). Data represent mean ± sem. Based on data presented in James *et al.* (2013).

Some studies on thermal acclimation indicate that the capacity to acclimate to temperature change varies between species and can vary between populations within a species, possibly determined by the long-term variability in temperature that a population or species is subjected to (Johnston and Temple, 2002; Angilletta, 2009; Seebacher *et al.*, 2012; Le Roy *et al.*, 2017). Phenotypic plasticity in response to temperature seems to be more common in ectotherms living in large bodies of water as daily changes in temperature are buffered by the water volume unlike any large, often gradual, seasonal changes in temperature (Johnston and Temple, 2002). Species with the capacity to acclimate in response to temperature change have, in some studies, been shown to alter the mechanical properties of skeletal muscle via mechanisms, such as changes in myofibrillar ATPase activity, differential expression of contractile protein isoforms, or changes in calcium handling (Johnston and Temple, 2002; Syme 2006). Such species that are able to acclimate may be better equipped to deal with, at least, small long-term changes in environmental temperature that can occur as a result of climate change. However, it is unclear whether these species can deal well with an overall more variable climate, particularly as such variability could mask the cue to acclimate.

### Dormancy

Avoidance of extreme temperature and drought can be achieved via undergoing periods of dramatically reduced metabolism, often referred to as ‘dormancy.’ The effects of periods of dormancy on the mechanical performance of skeletal muscle and locomotion have not been extensively studied. Periods of dormancy to avoid drought and extreme temperatures are termed aestivation and can extend up to many months or years (Pinder *et al.*, 1992).

Aestivation for 3 months did not affect burst swimming performance of the green-striped burrowing frog, *Cyclorana alboguttata* (Hudson and Franklin, 2002). In addition, 9 months of aestivation in the green-striped burrowing frog did not cause any significant changes in the maximal power output or fatigue resistance of isolated iliofibularis or sartorius muscles during work loop studies (Symonds *et al.*, 2007), despite significant reduction in the rate of tetanus force relaxation in iliofibularis muscle from aestivated individuals. Green-striped burrowing frogs are able to maintain skeletal muscle mass and cross-sectional area over these durations of dormancy (Symonds *et al.*, 2007; Mantle *et al.*, 2009), at least partially via reduced metabolism and increased expression of anti-apoptotic genes (Reilly *et al.*, 2013), enabling absolute muscle mechanical performance to be maintained ready for emergence from aestivation.

While climate change is more likely to cause regions to be on average warmer than colder, extreme weather events are likely to be more common and some of the overall findings from studies on hibernation may provide useful insight into dormancy in general. Up to 4 months of hibernation, while submerged in water, in the common frog, *Rana temporaria*, had no effect on maximal isometric force, force–velocity relationships from isovelocity experiments, or work loop power output in sartorius muscle (West *et al.*, 2006). In the 13-lined ground squirrel, *Ictidomys tridecemlineatus*, 3 months of hibernation did not cause a change in the maximal power output of soleus muscle (Fig. 6) but did reduce fatigue resistance during work loop studies (James *et al.*, 2013). Previous studies on ground squirrels have indicated little or no change in skeletal muscle fibre type but a decrease in skeletal muscle mitochondrial respiration rates, which would help explain the mechanical properties observed (James *et al.*, 2013). Many other hibernating mammals, such as bears, bats, and various rodents, undergo limited muscle atrophy, with no change in muscle fibre type or small shifts toward more oxidative fibres and little or no changes in the mechanical properties of skeletal muscle (Cotton, 2016). Figure 6.

These studies on hibernation reinforce the findings on aestivation to demonstrate that some species are able to spend substantial periods of time in a dormant state to avoid unfavourable climatic conditions without appreciable effects on subsequent mechanical performance of skeletal muscle, which is particularly important as post-dormancy such species tend to focus on fitness-related behaviours, such as finding and securing a mate. It is possible that further climate change could extend the periods of dormancy required. However, the possible duration of dormancy is limited, not least by the supply of stored energy required to maintain survival (Storey and Storey, 2007).

### Conclusions and future directions

The present review has demonstrated that changes in temperature can have profound effects on the mechanical performance of skeletal muscle, in turn affecting locomotor performance, behaviour, and potential survival. In some species acclimation, dormancy or other behavioural responses could reduce or remove potential effects of climate change on performance; there are limits to the efficacy of such approaches to dealing with climate change and many species are unable to acclimate or undergo dormancy. It is clear that the effects of temperature on muscle physiology differ between species and in some cases between populations within a species, which adds further complexity to modelling potential effects of climate change on fitness and species distribution. For example, the capacity of mosquitofish (*Gambusia holbrooki*) to acclimate critical sustained swimming performance and skeletal muscle metabolic enzyme activities has been found to vary between populations, even between populations from the same lineage (Seebacher *et al.*, 2012). However, some species from relatively stable thermal environments, such as the Antarctic fish *Pagothenia borchgrevinki*, seem unable to acclimate burst locomotor performance in response to environmental temperature change (Wilson *et al.*, 2001), although they are able to improve critical sustained swimming performance at higher temperatures via acclimation (Seebacher *et al.*, 2005). While thermal acclimation incurs energetic costs, it can in some cases enable performance to be improved across a season and may facilitate some species to better buffer against thermal effects of climate change. In contrast, other species may also cope well with thermal effects of climate change by having relatively low thermal sensitivity, which although this can entail a relatively lower performance, it would be maintained over a wider temperature range.

As discussed, individuals may respond to climate change by rapidly altering behaviours or timing of behaviours, such as mating, locomotion, food acquisition, habitat use, and predator avoidance (Beever *et al.*, 2017). For example, in response to higher temperatures, some animals use microhabitats that provide lower temperatures, as has been observed in fish, mammals, and reptiles. However, the success of such an approach depends on availability of, and competition for, such refuges and the ability of such animals to have sufficient time outside the refuge to undertake key fitness-related behaviours (Beever *et al.*, 2017).

While many journal papers considering effects of temperature on skeletal muscle and locomotor performance set such studies against a background of climate change, there are not enough data to indicate whether increases or decreases in environmental temperature, of the magnitude likely in climate change scenarios, will cause meaningful effects on skeletal muscle mechanics, locomotor performance, and behaviour in the species studied as few studies have estimated the likely remaining capacity of a population to buffer against such changes. Responses of a species to climate change would ideally also need to be considered in terms of their wider ecosystem to account for climate change effects on such aspects as predator–prey interactions (Gabriel *et al.*, 2005).

Further work needs to be done to better simulate the effects of prolonged locomotor activity in isolated muscle preparations to clarify the effects of temperature on sustained mechanical performance of skeletal muscle. As most previous studies have focused on thermal effects on one level of organization in a species removed from its environment, it is difficult to fully comprehend how important the broader effects of climate change on muscle mechanics will be in influencing local extinction rates of species. Therefore, additional work is needed to better understand the effects of temperature-induced changes on the mechanical performance of skeletal muscle on fitness-related whole animal performance, behaviour, and in turn the ability of a species to maintain its geographical range. Further work is also needed to investigate whether different climate change–related variables interact in their effects on skeletal muscle and whole animal performance, e.g. changes in ocean pH, habitat fragmentation, dehydration (more likely during periods of drought), and effects of pollutants (such as plastics, etc).
